# Investigation of the Cytotoxicity of Electrospun Polysuccinimide-Based Fiber Mats

**DOI:** 10.3390/polym12102324

**Published:** 2020-10-11

**Authors:** Kristof Molnar, Rita Varga, Benjamin Jozsa, Dora Barczikai, Eniko Krisch, Krisztina S. Nagy, Gabor Varga, Angela Jedlovszky-Hajdu, Judit E. Puskas

**Affiliations:** 1 Laboratory of Nanochemistry, Department of Biophysics and Radiation Biology, Semmelweis University, Nagyvarad ter 4, H-1089 Budapest, Hungary; molnar.182@osu.edu (K.M.); Rita.varga.94@gmail.com (R.V.); jozsa.benjamin93@gmail.com (B.J.); barczikai.dora@gmail.com (D.B.); s.nagykriszti@gmail.com (K.S.N.); 2 Department of Food, Agricultural and Biological Engineering, College of Food, Agricultural, and Environmental Sciences, The Ohio State University, 222 FABE, 1680 Madison Avenue, Wooster, OH 44691, USA; eniko2@krisch.hu; 3 Department of Oral Biology, Semmelweis University, Nagyvarad ter 4, H-1089 Budapest, Hungary; varga.gabor@dent.semmelweis-univ.hu

**Keywords:** polysuccinimide, plasma treatment, crosslinking, cytotoxicity, allylamine

## Abstract

This study investigated cell viability in the presence of allylamine-modified and plasma-treated electrospun polysuccinimide fiber mats (PSI-AAmp). Low pressure non-equilibrium plasma was used for crosslinking the PSI-AAm. Comparison of FTIR and XPS analyses demonstrated that crosslinking occurred on the surface of the samples. Cell viability was investigated using the MG-63 osteosarcoma cell line and WST-1 viability reagent. Since PSI hydrolyzes to poly(aspartic acid) (PASP), PASP was used in addition to the regular controls (cells only). Phase contrast showed normal morphology in all cases at 24 h; however, in the presence of PSI-AAmp at 72 h, some rounded, dead cells could also be seen, and proliferation was inhibited. Since proliferation in the presence of PASP alone was not inhibited, the cause of inhibition was not the final product of the hydrolysis. Further investigations will be carried out to pinpoint the cause.

## 1. Introduction

Polymeric nanostructures, including nanoparticles, micelles, nanofibers, or dendrimers, just to name a few, have become a focus of biomedical research in the past decades [1]. Such materials have helped to achieve more accurate and reliable diagnoses, effective targeting of drugs, and the revolution of regenerative medicine. However, polymeric materials have to fulfill several criteria in order to be applicable for biomedical purposes, tissue-compatibility being the most important among them [2,3,4]. Nanofibrous structures mimicking the human extracellular matrix (ECM) are promising candidates for application in tissue engineering and wound dressing [5]. Wound dressings aim to prevent further harm to the tissue, promote healing, and result in the best aesthetic repair [6,7]. The porous structure of polymer nanofibers ensures good oxygen permeation, absorption of wound discharge, and prevention of moisture loss. Furthermore, therapeutic agents, such as antibacterial or anti-inflammatory drugs can be incorporated into the fibrous material for more sustained release. Polymeric nanofibers for biomedical applications can be fabricated by a range of techniques, including phase separation, membrane templating, and self-assembly, but electrospinning is the most versatile method [8,9]. A large variety of polymers can be used for electrospinning, but the fibers need to be stable in an aqueous medium [10,11]. In the case of water-soluble polymers, crosslinking of the polymer is necessary so that the fibers can retain their integrity even in aqueous medium by forming polymer gel fibers [12,13,14]. Poly(aspartic acid) (PASP), and its anhydride polysuccinimide (PSI) are biodegradable, tissue-friendly materials, promising great potential in biomedical fields. PSI can be synthesized from aspartic acid by a solvent-free method, and its hydrolysis in alkaline medium results in water-soluble PASP [15,16]. PSI can be modified with a large variety of molecules due to the highly reactive succinimide rings; thus, PSI derivatives can be synthesized with various functional groups (e.g., thiol, alkyl, amine groups) [17,18,19]. PSI and its derivatives can be crosslinked with bifunctional molecules. The crosslinked PSI can then be hydrolyzed to yield PASP hydrogels for various biomedical applications [20,21,22]. However, only a few attempts to create crosslinked electrospun PSI and PASP nanofibers can be found in the literature. Zhang et al. synthesized crosslinked PSI fibers by immersing the electrospun nanofibers in a diamino-ethanol solution where the succinimide rings reacted with the primary amines. These fibers did not dissolve upon hydrolysis in alkaline medium, although their fibrous structure was partly lost due to the merging of the individual fibers [23]. Our research groups have also published some papers about the synthesis of crosslinked electrospun PSI mats. In the first case, co-axial electrospinning was used, where the core of the needle contained the diamine crosslinker, while the PSI was in the shell [24]. Crosslinked PSI fibers were successfully synthesized, although the fiber mat had to be washed thoroughly to remove the excess diamine. In the second case, thiol-modified PSI was electrospun, and disulfide crosslinks were created via air oxidation during the electrospinning process [16]. These fibers were found to be stable in water in vitro, although disulfide linkages can disintegrate under physiological conditions due to the presence of reducing agents in body fluids [25]. In the third case, crosslinking of allylamine-modified PSI-based fiber mat (PSI-AAm) was induced by plasma treatment [26]. The crosslinked structure was confirmed by the fact that the plasma-treated PSI-AAm (PSI-AAmp) did not dissolve in dimethyl sulfoxide, which is a good solvent for the non-crosslinked (non-plasma-treated) PSI-AAm. X-ray photoelectron spectroscopy (XPS) showed structural changes on the surface of the samples. PSI-AAmp were then hydrolyzed into PASP-AAmp, and SEM images demonstrated that the mats retained their fibrous structure. It was also shown that the plasma treatment sterilized the fiber mats. 

PSI- and PASP-based mats were shown to be non-cytotoxic. Gyarmati et al. published that 1,2-diaminobutane crosslinked PASP hydrogels were non-toxic to human intestinal epithelial cells (Caco-2) [27]. Németh et al. synthesized PASP derivatives with alkyl side groups, and none of the derivatives were found to be cytotoxic in the presence of human prostate cancer cells [28]. Furthermore, PASP hydrogels modified with Arg-Gly-Asp (RGD) tripeptide are biodegradable and can support survival, proliferation, and migration of human osteoblast-like cells (MG-63) [25]. The cytotoxicity of the PSI-AAmp and PASP-AAmp mats, however, has not yet been evaluated. 

Here, the investigation of the cytotoxicity of PSI-AAmp and PASP-AAmp fiber mats using MG-63 osteosarcoma cells is presented. 

## 2. Materials and Methods

### 2.1. Materials

L-aspartic acid (Sigma-Aldrich, Darmstadt, Germany), dimethylformamide (DMF) (VWR International, Radnor, PA, USA), dimethylsulfoxide (DMSO) (Sigma-Aldrich, Darmstadt, Germany), nutrient broth (Fluka Analytical, Charlotte, North Carolina, USA), o-phosphoric acid (VWR International, Radnor, PA, USA), phosphate buffer saline (PBS) (Sigma-Aldrich, Darmstadt, Germany), imidazole (ACS reagent, ≥99%, Sigma-Aldrich, Darmstadt, Germany), citric-acid*H2O (ACS reagent, ≥99.9%, VWR International, Radnor, PA, USA), sodium-chloride (99-100.5%, Sigma-Aldrich, Darmstadt, Germany), minimum essential medium (MEM) (Gibco, Waltham, MA, USA), fetal bovine serum (FBS, Gibco, Waltham, MA, USA), L-glutamine (Gibco, Waltham, MA, USA), non-essential amino acids (NEAA, Gibco, Waltham, MA, USA), penicillin-streptomycin mixture (Gibco, Waltham, MA, USA), trypsin/EDTA (Gibco, Waltham, MA, USA), 2-(4-Iodophenyl)-3-(4-nitrophenyl)-5-(2,4-disulfophenyl)-2H-tetrazolium (WST-1 cell proliferation reagent, Roche, Basel, Switzerland). All the chemicals were of analytical grade and used as received. For the aqueous solutions, ultrapure water (ZeneerPower I Water Purification System, Human Corporation, Seoul, South-Korea) was used.

### 2.2. Synthesis of Polysuccinimide (PSI), Poly(aspartic acid) (PASP) and Aallylamine-Modified Ppolysuccinimide (PSI-AA)

The synthesis of allylamine-modified polysuccinimide (PSI-AA) was published previously [16,26]. In this study, allylamine-modified PSI with a theoretical grafting degree (GD) of 2 (every second repeat unit had one AA attached) was used. The polymer was dialyzed against distilled water (cut off = 10 kDa) for 5 days, while the distilled water was changed 3 times on the first day, then once a day. Finally, the resulting PSI-AA was freeze-dried. 

### 2.3. Electrospinning of PSI-AA and Plasma Treatment

The electrospinning instrument consisted of a KD Scientific KDS100 syringe pump (Holliston, MA, USA), GENVOLT 73030P DC power supply (Bridgnorth, UK), and an aluminum plate collector placed at a distance of 15 cm in front of the electrospinning needle. 0.5 mL 45 w/w% PSI-AA/DMF solution was loaded into a 2.5 mL disposable plastic syringe and expelled at a rate of 1 mL/h through a G18 blunt end needle (Hamilton, Bonaduz, Switzerland). The voltage was set to 11 kV to maintain a stationary state during electrospinning. The fibrous membrane (PSI-AAm) was then removed from the collector, and small ~5 mm diameter disks were cut for the cell culture studies. The disks were placed in a Petri dish with a perforated Petri dish cover and were exposed to low-pressure non-equilibrium air plasma treatment using a Zepto benchtop plasma reactor (Diener Electronics GMBH, Ebhausen, Germany) [26]. The low frequency (40 kHz) plasma generator surrounded a 105 mm diameter, 300 mm depth tubular reactor with the samples. The chamber was evacuated to 0.3 mbar by a Pfeiffer Duo 1,6 vacuum pump (Asslar, Germany) while air (24 °C and 30–50% relative humidity) was continuously fed through a needle valve, and the pressure was kept at 0.3 mbar and treated for 17 min at 100 W power. Crosslinking was tested by submerging a sample into DMF. If the sample did not dissolve, the crosslinking was successful.

### 2.4. Scanning Electron Microscopy (SEM)

For SEM analysis, samples were placed on conductive carbon tape and sputter-coated using a 2SPI Sputter Coating System (West Chester, PA, USA) with 20–30 nm gold. Pictures were taken with a Zeiss Evo 40 XVP (Oberkochen, Germany) SEM (accelerating voltage 20 kV, magnification 5000). For fiber diameter analysis, 50 individual fibers were measured and analyzed by using the ImageJ software. 

### 2.5. Attenuated Total Reflection Fourier Transform Infrared Spectroscopy (ATR-FTIR)

ATR-FTIR was carried out using a JASCO FT/IR-4700 spectrophotometer (Tokyo, Japan) fitted with an attenuated total reflection (ATR) accessory (JASCO ATR Pro One). Spectra were collected in a range of (4000 cm^−1^–400 cm^−1^) with a resolution of 2 cm^−1^ with 126 scans. Spectra were corrected for H_2_O and CO_2_.

### 2.6. X-ray Photoelectron Spectroscopy (XPS)

Measurements were carried out using a Kratos Axis Ultra XPS (Manchester, UK) with a monochromated Al Kα source (1486.6 eV, 12 kV, 10 mA) and a charge neutralizer. Survey spectra were collected with 100 eV pass energy, while high resolution spectra were collected with 20 eV pass energy. The CasaXPS software was used for data analysis. The corresponding reference signal was the C1s signal with a binding energy of 285 eV. Curve fitting was performed using the Gaussian–Lorentzian distribution with the deduction of the Shirley background.

### 2.7. Cell Viability Assay

**Figure 1:**
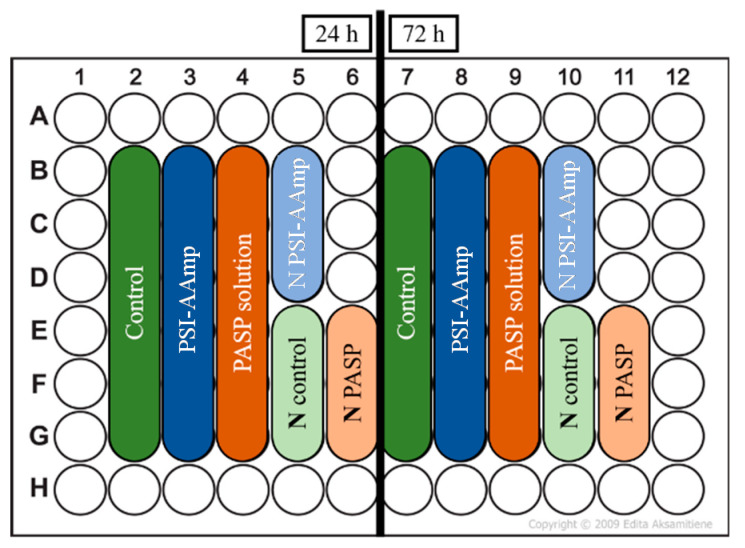
Sample distribution on the cell culture plate. **N** stands for negative control.

MG-63 osteosarcoma cells (Sigma-Aldrich, Darmstadt, Germany) were cultured in the following medium according to the manufacturer’s instructions: MEM supplemented with 10 % FBS, 2 mM L-glutamine, 1 % NEAA, 100 units/ml penicillin, and 100 mg/ml streptomycin. The cells were seeded into a 96-well cell culture plate with a concentration of 3700 cells/well in 100 μL culture medium (each well has a surface area of 0.37 cm^2^). Cells were incubated for 24 h in the plates to enable them to attach to the surface and proliferate. Then, PSI-AAmp mats and PASP solutions were added to the wells as shown in Figure 1. The control wells remained untreated; therefore, they contained only cell culture. Before the tests, PSI-AAmp mats were soaked in 300 ppm chlorine dioxide solution (a mixture of PBS buffer and Solvocid (Solumium LLC, Budapest, Hungary) in a ratio of 9:1) to ensure sterile conditions [29].

Viability tests were carried out for 24 h and 72 h. Cell viability was quantified by adding 100 μL WST-1 reagent (diluted 1:20 in MEM lacking Phenol Red and supplementation) to the wells and incubating them for 4 h at 37 °C. Then, the supernatants were transferred to a new plate and the light absorbance at 450 nm was measured in a microplate reader (Model 3550, Bio-Rad Laboratories, Tokyo, Japan). The morphology of the cells was monitored under an inverted phase-contrast microscope (Nikon Eclipse TS100, Nikon, Tokyo, Japan). Photomicrographs were taken with a 10x objective by a high-performance CCD camera (COHU, Poway, CA, USA) applying the Scion image software. For statistical evaluation of the viability data (N = 5 for each time point), the STATISTICA 10 software (TIBCO Software Inc., Palo Alto, CA, USA) was used to run Kruskal-Wallis (non-parametric) ANOVA and the median test. Values of *p* < 0.05 were considered statistically significant.

## 3. Results and Discussion

### 3.1. Electrospinning of PSI-AA and Crosslinking via Plasma Treatment

**Figure 2:**
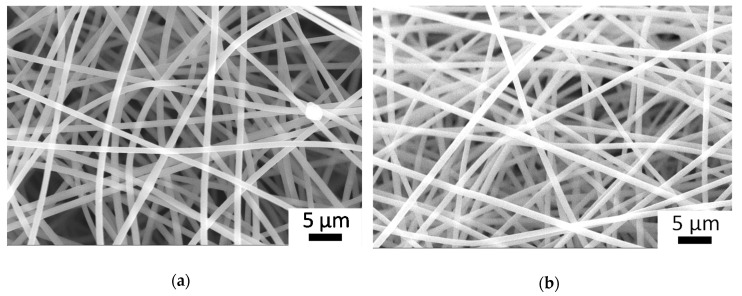
SEM image of electrospun allylamine-modified polysuccinimide fiber mat (PSI-AAm) (**a**) and the same after plasma treatment (PSI-AAmp) (**b**).

Figure 2 displays the SEM images of electrospun PSI-AAm (GD = 2) and PSI-AAmp. Figure 2a shows good quality fibers with no irregularities, similar to previous experiments. The diameter of the fibers showed a narrow size distribution with an average of 1000 ± 45 nm. After plasma treatment, there was no change in fiber morphology, and the average fiber size remained practically identical (1068 ± 35 nm, Figure 2b). Crosslinking was demonstrated by solubility testing. Plasma-treated and non-treated fiber mats were both immersed in DMF, a good solvent of PSI-AA. Non-plasma treated samples dissolved almost immediately, while plasma treated mats kept their integrity. 

**Figure 3:**
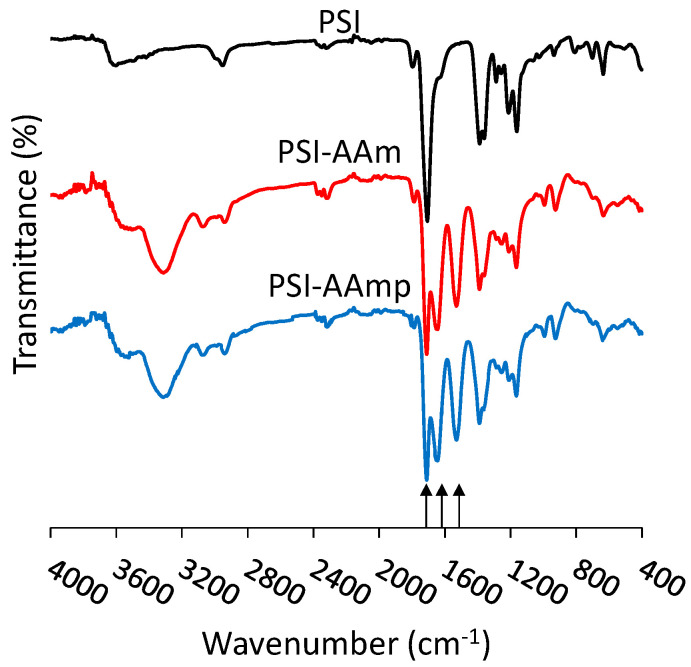
FTIR spectra of PSI, PSI-AAm and PSI-AAmp, where arrows from left to right indicate 1709, 1662, 1530 cm^−1^.

It was shown earlier that crosslinking occurred only on the surface by comparing FTIR and XPS data. The FTIR spectra of PSI-AAm and PSI-AAmp are compared in Figure 3. The characteristic absorption bands of imide rings can be seen at 1709 cm^−1^ (asymmetric stretching ν_C=O_ of C(=O)N), 1391 cm^−1^ (δ_C=O_ bending), and 1355 cm^−1^ (stretching ν_C–N_ of C(=O)N) [30]. In the case of PSI-AAm and PSI-AAmp, the peak at 1662 cm^−1^ (stretching ν_C=O_) indicates opened succinimide rings due to the reaction between allylamine and succinimide rings. The peak at 1530 cm^−1^ is the stretching vibration of the C=C double bond in the grafted allylamine, so it is absent in the spectrum of the PSI but present in both PSI-AAm and PSI-AAmp [31]. There is no difference between the FTIR spectra of PSI-AAm and PSI-AAmp, although a reduction of the 1530 cm^−1^ vibration is expected upon crosslinking. FTIR detects several tens of nanometers inside a sample even in the ATR mode, leading to a much bigger contribution of the bulk than the surface to the IR signal [32]. In contrast, X-ray photoelectron spectroscopy (XPS) at 90° probes ~10 nm from the surface and shows changes upon plasma treatment [26]. The survey spectra of PSI-AAm and PSI-AAmp had three major peaks representing C, N, and O atoms in both samples (not shown). The untreated sample had less N and O on the surface than the theoretical composition, indicating enrichment of non-polar groups facing the sample-air interface. The O content in the plasma-treated sample increased to 22.07 atomic % from 19.03 % in the non-treated sample (Table 1). This indicates O enrichment on the surface due to the plasma treatment. 

**Figure 4:**
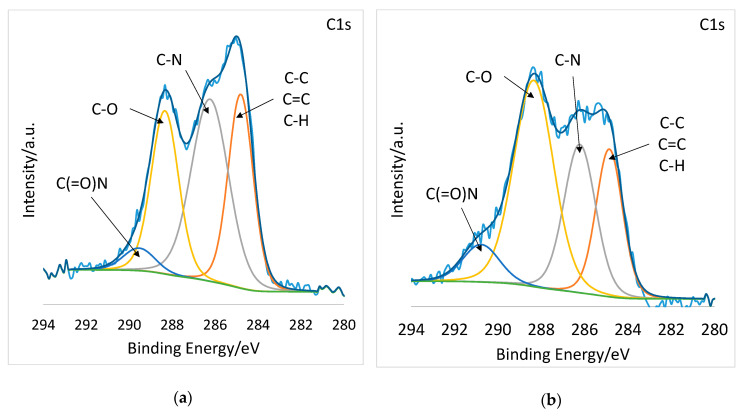

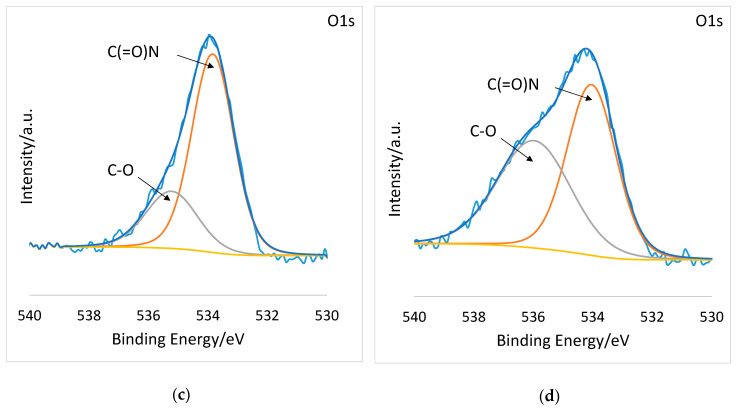
High resolution C1s and O1s spectra of PSI-AAm (**a**,**c**) and PSI-AAmp (**b**,**d**).

Most revealing are the high-resolution spectra. Figure 4a and b display the high resolution C1s spectra deconvoluted into C−C, C=C, and C−H at 285 eV, C−N at 286 eV, C−O at 288, and C(=O)N at 290 eV [33,34,35]. After plasma treatment, the C-O component grew considerably, from 24.27 % to 45.29 %, while the C−C, C=C, and C–H component decreased from 29.64 to 20.5 atomic % (Table 2). The high-resolution O1s spectra of PSI-AAm (Figure 4c) and PSI-AAmp (Figure 4d) are deconvoluted into two components: C(=O)N at 530 eV and C−O at 532 eV [36]. Again, the C–O component grew considerably from 28.45 % to 45.69 % (Table 2). This indicates that low-pressure non-equilibrium air plasma induced chemical changes on the surface, leading to crosslinking and thus insolubility in DMF.

### 3.2. Cell Morphology and Viability Study

**Figure 5:**
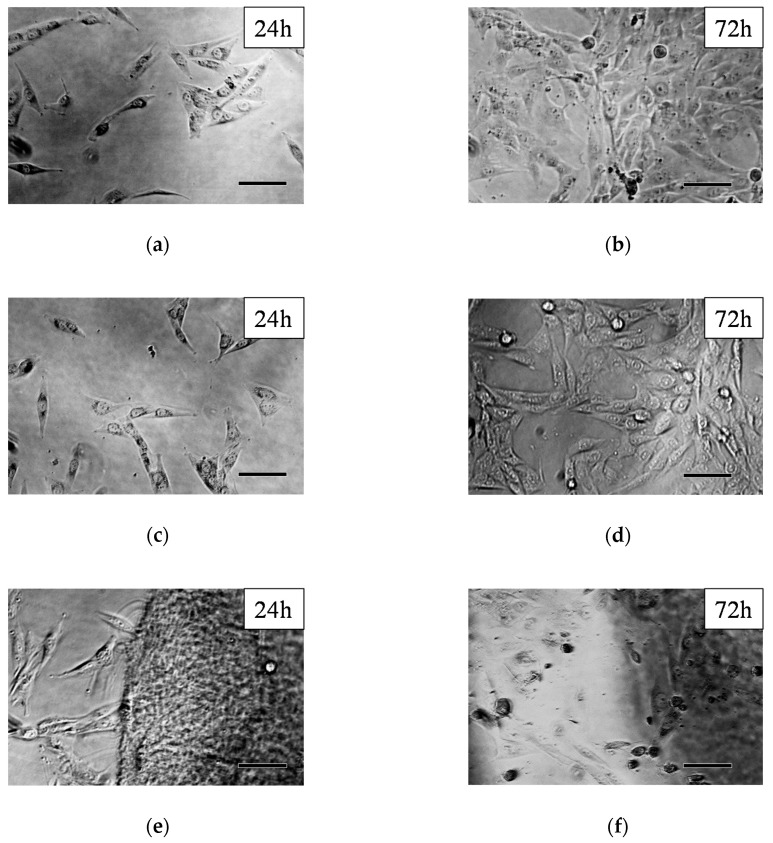
Phase-contrast microscopical images of MG-63 cells: controls after 24 (**a**) and 72 h (**b**), PASP control after 24 (**c**) and 72 h (**d**), and PSI-AAmp after 24 (**e**) and 72 h (**f**) incubation. Scale bars represent 100 µm.

Phase-contrast microscopic images of the MG-63 cells after 24 and 72 h after regular incubation (control), and with PASP control and PSI-AAm can be seen in Figure 5. The untreated control cells show their normal, fibroblast-like morphology at both time points (Figure 5a,b). After 72 h, the cells almost covered the whole bottom of the wells (Figure 5b). As it was described in the introduction, PSI undergoes hydrolysis to form PASP at pH 7.4; thus, control experiments were also carried out with PASP. Cells with normal morphology can be seen after 24 h (Figure 5c), and a large number of cells with normal, elongated morphology can be observed even after 72 h. The formation of PASP-AAmp hydrogel from PSI-AAmp could be observed visually as the mats swelled and became transparent in the aqueous cell culture media. Although the hydrolysis of PSI-AAmp shifts the pH of the culture medium (MEM) toward the acidic region (below 6.8), this effect was obviously not drastic enough to cause cellular damage. After 24 h incubation, cells with normal fibroblast-like morphology can be seen (Figure 5e). However, after 72 h incubation, only a smaller number of viable cells were found, and dead cells with a rounded shape can also be observed (Figure 5f). 

**Figure 6:**
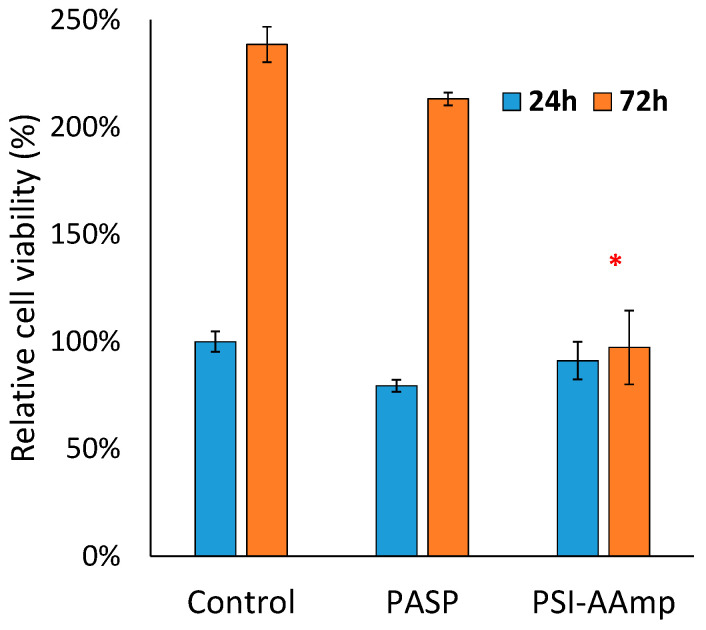
Relative viability of MG-63 cells 24 and 72 h after incubation with PASP or PSI-AAmp. The results were normalized to the UV absorbance of the 24 h control. The data are represented as arithmetic mean ± SEM (standard error of the mean). * significant difference (*p* < 0.05) compared to the control at the same time point.

The cell viability assay confirmed the results of the microscopical studies. The UV absorption of formazan forming in the mitochondria of viable cells after adding the WST-1 reagent is directly proportional to the number of the viable cells [25]. Figure 6 compares relative cell viability data in the presence (PSI-AAmp and PASP) and absence (control) of the test materials.

After 24 h, no significant difference in viability between groups was detected. Consequently, neither the PSI-AAmp samples (that had hydrolyzed into PASP-AAmp mats) (*p* = 0.64) nor the PASP (*p* = 0.06) itself caused changes in cellular viability within 24 h. After 72 h, the cultures exposed to PASP reached almost the same viability as the control (*p* = 0.29), while the cells exposed to plasma-treated and hydrolyzed PSI-AAmp showed significantly lower viability (*p* = 0.001). The reason for the lower cell number with PSI-AAmp after 72 h compared to control is unclear. It is likely not due to its hydrolysis to PASP-AAmp, since PASP alone did not alter viable cell number versus control significantly. It is hypothesized that PSI-AAmp directed cells towards differentiation, thereby decreasing proliferation, which would be a highly beneficial scaffold property [37,38]. Alternatively, apoptosis and the proliferation of cells may compensate each other, so viability remains unchanged between 24 and 72 h [37,38]. These possibilities have to be tested in further investigations.

## 4. Conclusions

It was previously shown that low-pressure non-equilibrium plasma can be used to induce crosslinking on the surface of allylamine-modified polysuccinimide electrospun fibers (PSI-AAm). Crosslinking prevents the dissolution of such mats in aqueous medium, such as biological fluids, and therefore makes the fibrous mats suitable for biomedical purposes, including wound dressings or artificial tissues. In this study, cell viability was investigated using the MG-63 osteosarcoma cell line in the presence of PSI-AAmp mats. Cell morphology studies showed normal morphology at 24 h; however, at 72 h, some rounded, dead cells could also be seen. Relative cell viability at 24 h in the presence of PSI-AAmp and PASP control were similar to the regular control, but by 72 h, proliferation was inhibited in the presence of PSI-AAmp. Since proliferation at 72 h in the presence of PASP was not inhibited, hydrolysis cannot be the only cause of the inhibition, and the decreased cell number must be due to an altered balance of proliferation, differentiation, and apoptosis. Further studies will be necessary to investigate this phenomenon.

## Figures and Tables

**Table 1:** XPS survey spectra of PSI-AAm and PSI-AAmp.

Atoms	Theoretical Composition At %	PSI-AAm At %	PSI-AAmp At %
**C**	61.11	66.41	63.10
**N**	16.67	14.56	14.83
**O**	22.22	19.03	22.07

**Table 2:** High-resolution C1s and O1s XPS spectra of PSI-AAm and PSI-AAmp.

Bonds	Binding Energy (eV)	PSI-AAm At %	PSI-AAmp At %
**C1s**			
**C–C, C=C, C–H**	285	29.64	20.05
**C–N**	286	39.30	26.54
**C–O**	288	24.27	45.29
**C(=O)N**	290	6.79	8.12
**O1s**			
**C(=O)N**	530	71.55	54.31
**C–O**	532	28.45	45.69
